# Supplementary figures and images for: Evaluation of Next-Generation Sequencing Applied to Cryptosporidium parvum and Cryptosporidium hominis Epidemiological Study
Source: Pathogens. 2022 Aug 18;11(8):938. doi: 10.3390/pathogens11080938 (PMC9414878; doi:10.3390/pathogens11080938)

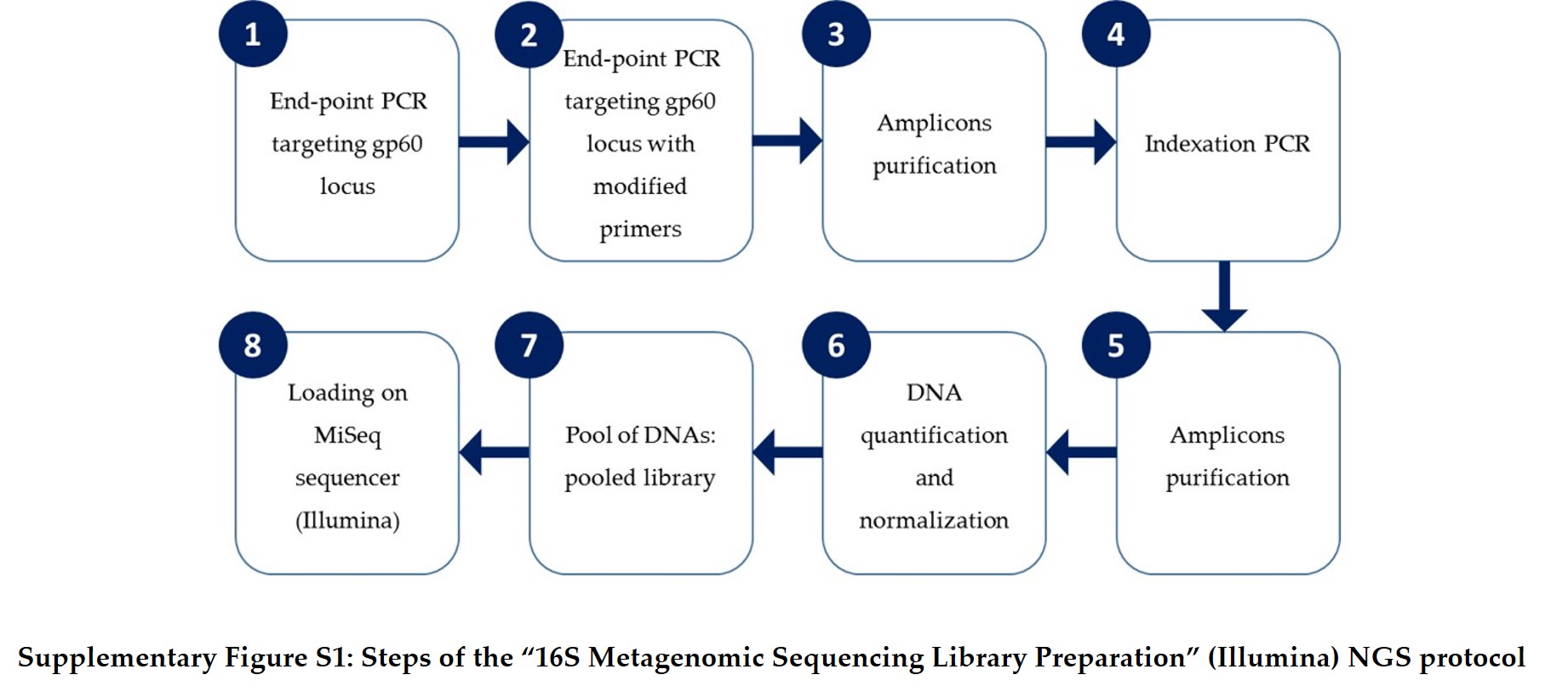

Supplement: Supplementary file 1 [file pathogens-11-00938-s001.zip › Figure S1.jpg]
